# A new radiological classification for the risk assessment of anterior skull base injury in endoscopic sinus surgery

**DOI:** 10.1038/s41598-020-61610-1

**Published:** 2020-03-12

**Authors:** Baharudin Abdullah, Shiun Chuen Chew, Mohd Ezane Aziz, Norasnieda Md Shukri, Salina Husain, Sng Weirong Joshua, De Yun Wang, Kornkiat Snidvongs

**Affiliations:** 1 Department of Otorhinolaryngology-Head & Neck Surgery, School of Medical Sciences, Universiti Sains Malaysia, 16150 Kubang Kerian, Kelantan Malaysia; 2 Department of Radiology, School of Medical Sciences, Universiti Sains Malaysia, 16150 Kubang Kerian, Kelantan Malaysia; 3 Department of Otorhinolaryngology- Head & Neck Surgery, Universiti Kebangsaan Malaysia Medical Centre, Jalan Yaacob Latif, Bandar Tun Razak, 56000 Kuala Lumpur Malaysia; 4 Department of Otolaryngology, Yong Loo Lin School of Medicine, National University of Singapore, Singapore, 119228 Singapore; 5 Department of Otolaryngology Head and Neck Surgery, Faculty of Medicine, Chulalongkorn University and King Chulalongkorn Memorial Hospital, 10330 Bangkok, Thailand

**Keywords:** Computed tomography, Risk factors

## Abstract

Keros and Gera classifications are widely used to assess the risk of skull base injury during endoscopic sinus surgery. Although, both classifications are useful preoperatively to stratify risk of patients going for surgery, it is not practical to measure the respective lengths during surgery. In this study, we aimed to propose a new radiological classification (Thailand-Malaysia-Singapore (TMS)) to assess the anatomical risk of anterior skull base injury using the orbital floor (OF) as a reference. A total of 150 computed tomography images of paranasal sinuses (300 sides) were reviewed. The TMS classification was categorized into 3 types by measuring OF to cribriform plate and OF to ethmoid roof. Most patients were classified as TMS type 1, Keros type 2 and Gera class II, followed by patients classified as TMS type 3, Keros type 1 and Gera class 1. TMS has significant correlation with Keros classification (p < 0.05). There was no significant correlation between Keros and Gera classifications (p = 0.33) and between TMS and Gera classifications (p = 0.80). The TMS classification has potential to be used for risk assessment of skull base injury among patients undergoing ESS. It serves as an additional assessment besides the Keros and Gera classifications.

## Introduction

Endoscopic sinus surgery (ESS) has an overall complication rate of 0.5% with the specific complications of cerebrospinal fluid leak, orbital injury, haemorrhage requiring surgery, blood transfusion and toxic shock syndrome at 0.09%, 0.09%, 0.10%, 0.18%, and 0.02%, respectively^1^. Understanding the computed tomography of paranasal sinus (CT PNS) variations in every patient and equipping oneself with the diverse anatomical knowledge is a prerequisite prior to surgery. The frequency of these variations may differ among different populations, but the orbital floor (OF) is always in constant position relative to the skull base that slopes posteriorly^2–5^. The OF or the medial maxillary sinus roof is known as an important intraoperative reference point to ensure a safe dissection and entry to the posterior sinuses (posterior ethmoid and sphenoid sinuses)^3–6^. It is a useful landmark when the normal anatomical structures are distorted by tumour or previous surgery.

Most people use the Keros classification^7^ to assess the length of the lateral lamella and the risk of skull base injury. Patients with a deep cribriform plate (CP) or Keros type 3 are more prone to skull base injury at the cribriform plate or the lateral lamella^8^. However, the Keros classification has limitations in describing the risk of intracranial entry due to the sloping shape of the skull. Subsequently, the Gera classification^9^ was proposed to consider the sloping level of ethmoidal roof (ER) relative to CP. By measuring the angle formed by the lateral lamella of CP and the continuation of the horizontal plane passing through CP, the risk of intracranial entry was divided into 3 classes; class I (>80 degrees, low risk), class II (45 to 80 degrees, medium risk) and class III (<45 degrees, high risk)^9,10^.

Although, both classifications are useful preoperatively to stratify risk of patients going for surgery, it is not practical to measure the respective lengths during surgery. When dissections are being done lateral to the middle turbinate, it is not easy to identify and define the lateral lamella and there is a risk of penetrating the skull base at this area. A new radiological classification is required to complement the other existing classification systems and provide a guide to surgeons on the suitable micro instruments for dissection at high risk area during ESS. The aims of the study were to propose a new radiological classification to assess the risk of anterior skull base injury using the OF as a reference and compare it with the Keros and Gera classifications.

## Materials and Methods

### Study setting and participants

The study protocol was reviewed and approved by the Human Research Ethics Committee of Universiti Sains Malaysia (Ethical Approval Code: USM/JEPeM/16090342), performed in adherence with the Declaration of Helsinki and approved guidelines. Written informed consent was obtained from all patients before their participation in this study. All available CT PNS of subjects 18 years and above were retrieved from the Radiology Information System (RIS) and Picture Archive Communication System (PACS) from 9^th^ January 2017 until 8^th^ January 2018. The inclusion criteria were CT PNS with slice thickness of 1 mm and capable of multiplanar reconstruction (MPR). The exclusion criteria were previous surgery to paranasal sinuses and skull base, previous skull base or facial trauma, previous surgery to ethmoid or sphenoid sinuses, sinonasal malignancy, severe rhinosinusitis with or without nasal polyps, chronic maxillary atelectasis with significant OF changes and craniofacial abnormalities.

### Radiological measurements

The CT PNS images were collected from computers at the workstation with 2 Mega Pixel monitor (Barco MPG 2121 monitor– resolution 2048 × 1536) via Picture Archive Communication System (PACS) in PACS Universal Viewer Version 5.0 SP6. The CT scan was performed with Siemens SOMATOM Definition AS+ on supine patient with collimation of 64 × 0.6, exposure of 135 kV and 200 mAs, 1 mm slice thickness and rotation time of 1.0 s. The bone view images were used for measurement. The measurements were done by three of the authors (CSC, MEA and BA). All measurements were taken 3 times and the average was used in the data analysis. When there was discordant opinion, further evaluation of the image was done to obtain a mutual consensus. The nasal floor is considered as the reference plane to measure all the other landmarks^5^.

### TMS classification

**Figure 1:**
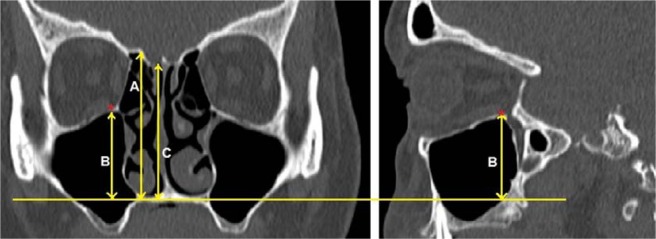
The heights of the ethmoid roof (**A**), orbital floor (**B**), and cribriform plate (**C**) relative to the nasal floor in coronal CT PNS. Each vertical height was measured 90° perpendicular to the nasal floor. To assess the risk of skull base injury using TMS classification, the distances from orbital floor to cribriform plate (OF-CP) and from orbital floor to ethmoid roof (OF-ER) were used. Red asterisk denotes the maximum height of the maxillary sinus which is the level taken as the orbital floor landmark.

**Figure 2:**
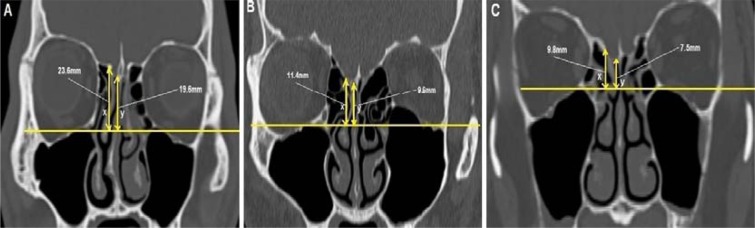
CT PNS coronal section showing the three types of TMS classification; (**A**) is Type 1 (low risk) where both OF-CP and OF-ER are 10 mm and above, (**B**) is Type 2 (moderate risk) where either OF-CP or OF-ER is less than 10 mm and (**C**) is type 3 (high risk) where both OF-CP and OF-ER are less than 10 mm.

Multiplanar (MPR) CT images were reconstructed to obtain the OF measurement in true horizontal plane in both the coronal and sagittal views. A line drawn along the nasal floor served as the reference point. In the coronal view, the right orbital floor was measured at the point where the right medial wall of the maxillary sinus roof was seen at its maximum height. In the coronal view (Fig. 1), the heights from nasal floor to right ER (labelled A), from nasal floor to right OF (a single view at the maximum height to the right maxillary sinus) (labelled B), and from nasal floor to right CP (labelled C) were measured. Each vertical height was measured 90° perpendicular to the nasal floor. When there was a lower cribriform height at another point anterior to the sphenoid (based on sagittal view), then this was used for the CP height. The measurements were repeated on the left. To assess the risk of skull base injury using the TMS classification, the distances from OF to CP (OF-CP) and from OF to ER (OF-ER) were used. We classified the risk of skull base injury into type 1, type 2 and type 3 (Fig. 2). Type 1 (low risk) is both OF-CP and OF-ER are 10 mm and above, or more than twice the depth of thru-cutting forceps when ESS is considered as safe, type 2 (moderate risk) is when either OF-CP or OF-ER is less than 10 mm, or less than twice of the depth of thru-cutting forceps when ESS should proceed with caution and type 3 (high risk) is both OF-CP and OF-ER are less than 10 mm or less than the depth of thru-cutting forceps when ESS should proceed with extreme caution.

### Keros classification

**Figure 3:**
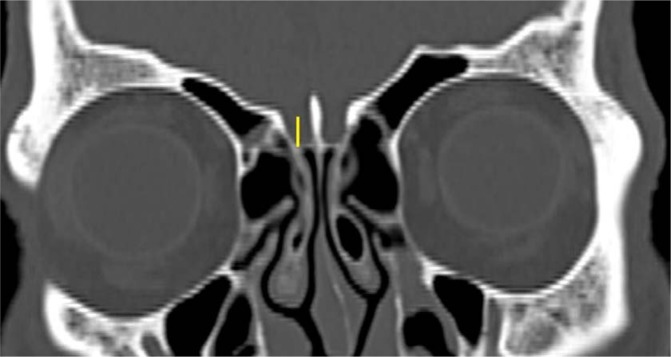
Keros classification^7^ was assessed according to the depth of the cribriform plate, measured as the vertical height of the olfactory fossa in the CT coronal plane and classified as type 1, 2 and 3.

The depth of the CP, measured as the vertical height of the olfactory fossa in the coronal plane on each side (Fig. 3), was measured and classified as type 1 (1 to 3 mm depth), type 2 (4 to 7 mm depth) or type 3 (more than 7 mm depth). Asymmetry in the depth (difference of more than 3 mm) between the right and left CPs was also recorded. The measurements were repeated on the left.

### Gera classification

**Figure 4:**
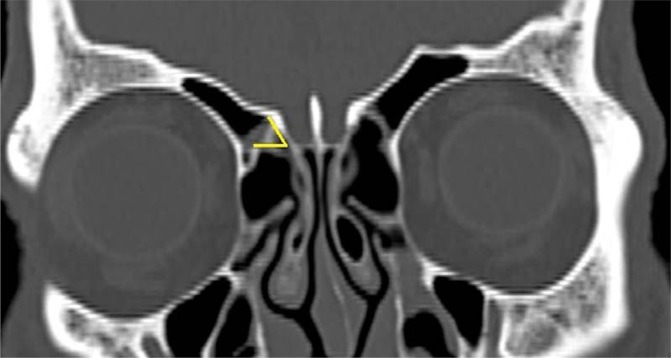
Gera classification^9^ was assessed by measuring at the angle formed by the lateral lamella of the cribriform plate and the continuation of the horizontal plane passing through the cribriform plate in the CT coronal plane and classified as Class I, II and III.

The Gera classification was classified into 3 classes depending on its amplitude and on the hypothetical risk of iatrogenic injuries: class I (>80 degrees, low risk), class II (45 to 80 degrees, medium risk) and class III (<45 degrees, high risk). Measurement was done at the angle formed by the lateral lamella of the CP and the continuation of the horizontal plane passing through the CP (Fig. 4). The measurements were repeated on the left.

### Statistical analysis

Descriptive parametric data were presented as percentage and mean (standard deviation [SD]). The correlations between the depth of cribriform, distance of OF-ER, distance of OF-CP, and angle formed by the lateral lamella of CP and the continuation of the horizontal plane passing through CP, were estimated using Pearson’s correlation coefficient (r). Chi Square test was used to assess the relationships between TMS, Keros and Gera classifications. A p value of <0.05 was considered statistically significant. The intraclass correlation coefficient test was used to assess the inter-rater reliability. Statistical analysis was performed using SPSS software version 22.0.

## Results

### Demographic data

A total of 150 CT PNS (300 sides) was evaluated. The subjects consist of 38% women with age ranged from 19 to 82 years (mean 56.2) and 62% male patients, age ranged from 18 to 89 years (mean 52.3). A total of 300 sides were measured independently (left and right sides). The degree of agreement among raters was excellent with inter-rater reliability at 0.99 (95% confidence interval, 0.98–0.99, p < 0.05).

### TMS classification

The OF was lower than the cribriform, ER and sphenoid roof in all cases (300 sides). The mean distance of the CP, ER and sphenoid roof to OF was 10.7 ± 2.1 mm, 14.1 ± 2.9 mm and 10.5 ± 1.8 mm respectively. 88.7% had type 1 (low risk), 1.6% had type 2 (moderate risk) and 9.7% had type 3 (high risk) according to TMS classification (Table 1). There was no significant difference in the distribution of TMS classification among males and females (p = 0.199).

**Table 1:** The different risk of skull base injury using TMS classification.

Type^*^	*Right* n (%)	*Left* n (%)	*Total* n (%)	Risk of skull base injury
1	132 (44)	134 (44.7)	266 (88.7)	Low
2	3 (1)	2 (0.6)	5 (1.6)	Moderate
3	15 (5)	14 (4.7)	29 (9.7)	High
Total	150	150	300	

^*^Type 1 is both OF-CP and OF-ER are longer than 10 mm, Type 2 is when either OF-CP or OF-ER is less than 10 mm, Type 3 is both OF-CP and OF-ER are less than 10 mm.

### Keros classification

The mean depth of the CP was 3.4 ± 0.8 mm (range, 1.0–6.9 mm). The most common was Keros type 2 (75.7% of cases), followed by Keros type 1 (24.3% of cases) but there was no Keros type 3. There was no significant difference in the distribution of Keros classification among males and females (p = 0.16). Asymmetry in the depth of the CP was seen in 26% of cases.

### Gera classification

The mean degree of the angle formed by the lateral lamella of the CP and the continuation of the horizontal plane passing through the CP was 70.1 ± 13.1° (range 28–88°). The degree of angle ranged from 45 to 80 degrees (class II, medium risk) in 72.3%, more than 80 degrees (class I, low risk) in 23.7% and less than 45 degrees (class III, high risk) in 4.0% of cases. There was no significant difference in the angle classification among males and females (p = 0.19).

### Comparison of skull base classifications

The depth of the cribriform has a strong positive correlation with OF-ER (r = 0.86) and OF-CP (r = 0.76), p < 0.05 (Table 2). The degree of angle formed by the lateral lamella of the CP and the continuation of the horizontal plane passing through the CP has a weak positive correlation with the depth of cribriform (r = 0.16), OF-ER (r = 0.17) and OF-CP (r = 0.16), p < 0.05. Most patients were classified as TMS type 1, Keros type 2 and Gera class II, followed by patients classified as TMS type 3, Keros type 1 and Gera class 1. There was a significant correlation between TMS and Keros classifications (p < 0.05) (Table 3) but no significant correlation between Gera and TMS classifications (p = 0.804) (Table 4) as well as between Gera and Keros classifications (p = 0.334) (Table 5).

**Table 2:** The correlations between the depth of the cribriform, orbital floor to ethmoid roof height, orbital floor to cribriform plate height, and the angle formed by the lateral lamella of the cribriform plate and the continuation of the horizontal plane passing through the cribriform plate (assessed by the Pearson’s correlation coefficient, r).

	Depth of the cribriform	OF-ER^*^	OF-CP^**^	Angle
Depth of the cribriform	1	0.86	0.76	0.16
OF-ER^*^	0.86	1	0.96	0.17
OF-CP^**^	0.76	0.96	1	0.16
Angle	0.16	0.17	0.16	1

^*^OF-ER- distance from orbital floor to ethmoid roof.

^**^OF-CP- distance from orbital floor to cribriform plate.

**Table 3:** The distribution of TMS (Thailand-Malaysia-Singapore) and Keros classifications^7^.

TMS*	Keros **			TOTAL (n)
	Type 1 n (%)	Type 2 n (%)	Type 3 n (%)	
Right				
Type 1	21 (14)	111 (74)	0	132
Type 2	0	3 (2)	0	3
Type 3	15 (10)	0	0	15
Left				
Type 1	23 (15.3)	111 (74)	0	134
Type 2	1 (0.7)	1 (0.7)	0	2
Type 3	13 (8.6)	1 (0.7)	0	14
TOTAL (n)	73	227	0	300

TMS and Keros^7^ classifications have significant correlation (p < 0.05).

*TMS (type 1: both OF-CP and OF-ER are longer than 10 mm, type 2: either OF-CP or OF-ER is less than 10 mm, type 3: both OF-CP and OF-ER are less than 10 mm).

^**^Keros (type 1: 1–3 mm depth of cribriform plate, type 2: 4–7 mm depth of cribriform plate, type 3: more than 7 mm depth of cribriform plate).

**Table 4:** Distribution of Gera^9^ and TMS (Thailand-Malaysia-Singapore) classifications.

Gera *	TMS**			TOTAL (n)
	Type 1 n (%)	Type 2 n (%)	Type 3 n (%)	
*Right				
Class I	35 (23.3)	2 (1.3)	2 (1.3)	39 (25.9)
Class II	90 (60)	1 (0.7)	12 (8)	103 (68.7)
Class III	7 (4.7)	0	1 (0.7)	8 (5.4)
**Left				
Class I	28 (18.6)	0	4 (2.7)	32 (21.3)
Class II	103 (68.7)	2 (1.3)	9 (6)	114 (76)
Class III	3 (2)	0	1 (0.7)	4 (2.7)
TOTAL (n)	266	5	29	300

Gera^9^ and TMS classifications have no significant correlation (p = 0.804).

^*^Gera (Class I, low risk: the angle >80 degrees, Class II, medium risk: the angle ranged from 45 to 80 degrees, Class III, high risk: the angle <45 degrees).

***TMS (type 1: both OF-CP and OF-ER are longer than 10 mm, type 2: either OF-CP or OF-ER is less than 10 mm, type 3: both OF-CP and OF-ER are less than 10 mm).

**Table 5:** The distribution of Gera^9^ and Keros^7^ classifications.

Gera^*^	Keros **			TOTAL (n)
	Type 1 n (%)	Type 2 n (%)	Type 3 n (%)	
Right				
Class I	9 (6)	30 (20)	0	39
Class II	24 (16)	79 (52.7)	0	103
Class III	3 (2)	5 (3.3)	0	8
Left				
Class I	9 (6)	23 (15.4)	0	32
Class II	26 (17.3)	88 (58.7)	0	114
Class III	2 (1.3)	2 (1.3)	0	4
TOTAL (n)	73	227	0	300

Gera^9^ and Keros^7^ classifications have no significant correlation (p = 0.334).

^*^Gera (Class I, low risk: the angle >80 degrees, Class II, medium risk: the angle ranged from 45 to 80 degrees, Class III, high risk: the angle <45 degrees).

^**^Keros (type 1: 1–3 mm depth of cribriform plate, type 2: 4–7 mm depth of cribriform plate, type 3: more than 7 mm depth of cribriform plate).

## Discussion

The ethmoid bone consists of the CP in the midline and the paired ethmoid sinuses laterally^11^. The ethmoid sinuses are bordered by the lamina papyracea laterally and the superior and middle turbinates medially. The ethmoid sinuses are separated from the anterior cranial fossa by the fovea ethmoidalis or ER. The ER is lowest at its medial aspect where it articulates with the CP and sloping gradually upwards at its lateral aspect^11^. The angle that it articulates with CP determines the vertical length of lateral lamella and where there is a difference in right and left side contributes to the asymmetry in the ER configuration. In a radiological study by Skorek *et al*.^12^, they found that Keros classification alone is not enough to identify the high-risk area at the skull base and the “dangerous ethmoids’. They recommended a classification that is based on the distance of olfactory fossa to both medial wall of orbit and the medial wall of middle turbinate as most ESS dissection occurs lateral to the middle turbinate. Furthermore, they opined more attention should be given to the bony margin of orbit and ER which comprise the anterior cranial fossa that is at risk of iatrogenic complications.

Intraoperatively, surgeons need a classification that is more practical for them to use. From studies conducted^4–6,13^, there was an average of at least 10 mm vertical distance from OF to the critical anatomy, namely; the CP, ER and sphenoid roof. This distance encompasses the bite size of many commonly used endonasal surgical instruments, such as the up through biting forceps, the mushroom punch, Kerrison Rongeurs, curette and non-biting up forceps, which have the depth of bite: 7 mm, 3 mm, 3 mm, 4 mm and 10 mm respectively^4,5^. We used the measurement of 10 mm as a dividing point for low, moderate and high risk as it represents more than twice of the depth of most instruments used during ESS. Using OF as a reference point, our classification can identify high risk patients for skull base injury whereby surgeons should use thru-cutting forceps with caution when dissecting in the high-risk group. Therefore, the TMS classification has potential benefits for surgeons to assess the risk of skull base injury among their patients undergoing ESS and guide their dissections in high risk patients. Moreover, by using this classification together with Keros and Gera classifications, surgeons will have a better assessment of the risk of intracranial injury preoperatively and intraoperatively as their guide towards safer dissection at high risk area.

Although, TMS classification has a significant correlation with the Keros classification, there is an interesting difference. We found some of our patients had type 3 TMS which is deemed as high risk of skull base injury even though they only have Type 1 and Type 2 Keros. There are different heights of ethmoidal roof in relation to orbital height^13^ and these are not reflected by the Keros classification. Therefore, TMS when used together with Keros classification, provides additional information to reduce the risk of skull base injury. The basis for the Gera classification is that a more pronounced slope of the anterior skull base may predispose to injuries of the medial part of the skull base when dissecting at medial ethmoidal cells and frontal sinus. At those areas, the instruments are close to the lateral lamella of the cribriform plate and it is possible that a more pronounced ethmoidal roof slope may predispose surgeons to inadvertent skull base violation when performing dissection from posterior to anterior. Although in their study^9^, the authors found Gera classification has significant positive correlation with the depth of CP, our data showed there was no significant correlation between Gera and Keros classifications. The TMS classification also has no significant correlation with Gera classification. There might be anatomical variations among different populations as our study is on Asians and their study is on Europeans which might explain this discrepancy. The different configuration of the sloping ethmoid roof between Asians and Europeans could also contribute to the discrepancy. Its further highlights that we need additional classification besides the currently available ones.

## Conclusions

The TMS classification is a new radiological classification that has potential to be used for risk assessment of skull base injury among patients undergoing ESS and as a guide for dissections at high risk areas. It serves as an additional assessment besides the Keros and Gera classifications. Future studies are required to validate this classification as a predictor of anterior skull base injury.

## Data Availability

The datasets generated during and/or analysed during the current study are available from the corresponding author on reasonable request.
